# Rhabdomyolysis: a genetic perspective

**DOI:** 10.1186/s13023-015-0264-3

**Published:** 2015-05-02

**Authors:** Renata Siciliani Scalco, Alice R Gardiner, Robert DS Pitceathly, Edmar Zanoteli, Jefferson Becker, Janice L Holton, Henry Houlden, Heinz Jungbluth, Ros Quinlivan

**Affiliations:** MRC Centre for Neuromuscular Diseases and Department of Molecular Neuroscience, University College London (UCL) Institute of Neurology and National Hospital for Neurology and Neurosurgery, London, UK; Department of Neurology, HSL, Pontifícia Universidade Católica do Rio Grande do Sul (PUCRS), Porto Alegre, Rio Grande do Sul Brazil; CAPES Foundation, Ministry of Education of Brazil, Brasilia, DF Brazil; Department of Basic and Clinical Neuroscience, Institute of Psychiatry, Psychology and Neuroscience, King’s College London (KCL), London, UK; Department of Neurology, School of Medicine, Universidade de São Paulo (FMUSP), São Paulo, SP Brazil; Department of Paediatric Neurology, Evelina Children’s Hospital, Guy’s & St Thomas NHS Foundation Trust, London, UK; Randall Division for Cell and Molecular Biophysics, Muscle Signalling Section, King’s College London, London, UK; Dubowitz Neuromuscular Centre, Great Ormond Street Hospital, London, UK

**Keywords:** Rhabdomyolysis, Myoglobinuria, Neuromuscular disorders, Diagnosis, Genetic, Polymorphism, Increased CK, Triggers, Phenotype, Gene, Muscle metabolism, Muscle Biopsy, Pathology

## Abstract

Rhabdomyolysis (RM) is a clinical emergency characterized by fulminant skeletal muscle damage and release of intracellular muscle components into the blood stream leading to myoglobinuria and, in severe cases, acute renal failure. Apart from trauma, a wide range of causes have been reported including drug abuse and infections. Underlying genetic disorders are also a cause of RM and can often pose a diagnostic challenge, considering their marked heterogeneity and comparative rarity.

In this paper we review the range of rare genetic defects known to be associated with RM. Each gene has been reviewed for the following: clinical phenotype, typical triggers for RM and recommended diagnostic approach. The purpose of this review is to highlight the most important features associated with specific genetic defects in order to aid the diagnosis of patients presenting with hereditary causes of recurrent RM.

## Abstract in Portuguese

A rabdomiólise (RM) é um evento agudo e grave, caracterizado por danos do músculo esquelético com a liberação, em grande quantidade, de componentes intracelulares para a corrente sanguínea. Uma vasta gama de causas tem sido relatada, incluindo trauma, abuso de drogas e infecções. Doenças hereditárias também podem causar RM, mas muitas vezes representam um desafio diagnóstico, considerando a sua heterogeneidade e raridade. Por fim, diversas doenças neuromusculares costumam estar associadas com níveis de CK cronicamente elevados, dificultando a identificação correta dos episódios de RM.

Nesse artigo, revisamos os diversos defeitos genéticos associados à RM. Cada gene foi revisado abrangendo os seguintes: fenótipo clínico, gatilhos para RM e abordagem diagnóstica. O objetivo desta revisão é destacar as características mais importantes associados a defeitos genéticos específicos, a fim de auxiliar o diagnóstico de pacientes com causas hereditárias de RM recorrente.

## Introduction

**Figure 1:**
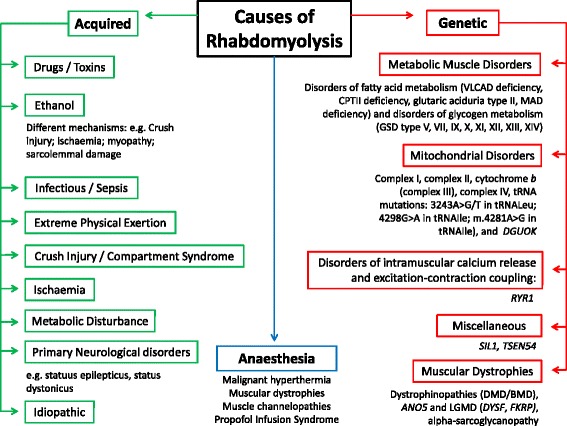
Examples of conditions associated with RM. In individual cases both genetic and environmental factors may combine to trigger a RM event; anaesthesia-induced RM is the best characterized example. VLCAD: very long-chain acyl-CoA dehydrogenase, CPTII: carnitine palmitoyl-transferase-II, MAD: multiple acyl-CoA dehydrogenase, GSD: glycogen storage disease, tRNA: Transfer Ribonucleic Acid, *DGUOK*: deoxyguanosine kinase gene, *RYR1*: Ryanodine Receptor 1 gene, *SIL1*: SIL1, S. Cerevisiae, homolog of, *TSEN54:* tRNA splicing endonuclease 54 gene, S. cerevisiae, homolog of, DMD: Duchenne Muscular Dystrophy, BMD: Becker Muscular Dystrophy, *ANO5*:Anoctamin 5 gene, LGMD: Limb-girdle Muscular Dystrophy, *DYSF*: Dysferlin gene, *FKRP:* fukutin-related protein gene [1,2,4,8,62,110-113].

Rhabdomyolysis (RM) is characterised by acute and often severe skeletal muscle damage resulting in the release of intracellular muscle components into the blood stream frequently resulting in myoglobinuria and, in severe cases, acute renal failure. Diverse etiologies (Figure 1) implicated in acute RM share a common final pathway, increased intracellular free ionized calcium, leading to muscle cell death through the activation of a number of detrimental mechanisms such as enzymatic activation and prolonged muscle fibre contraction [1,2]. Different genetic defects causing various neuromuscular and metabolic disorders are known to be associated with RM. Recurrent RM, exercise related complaints and a positive family history are common features of an underlying genetic condition. In some instances, RM may be due to a combination of genetic predisposition and environmental causes. In these cases a purely environmental factor may be considered the sole cause for the acute event with a relatively high risk of recurrence if the genetic diagnosis is not considered. Recently reported examples are a number of patients with “virally-induced” RM who in fact had malignant hyperthermia susceptibility (MHS)-associated *RYR1* mutations, resulting in a genetic predisposition for the virally-triggered muscle breakdown [3,4].

**Figure 2:**
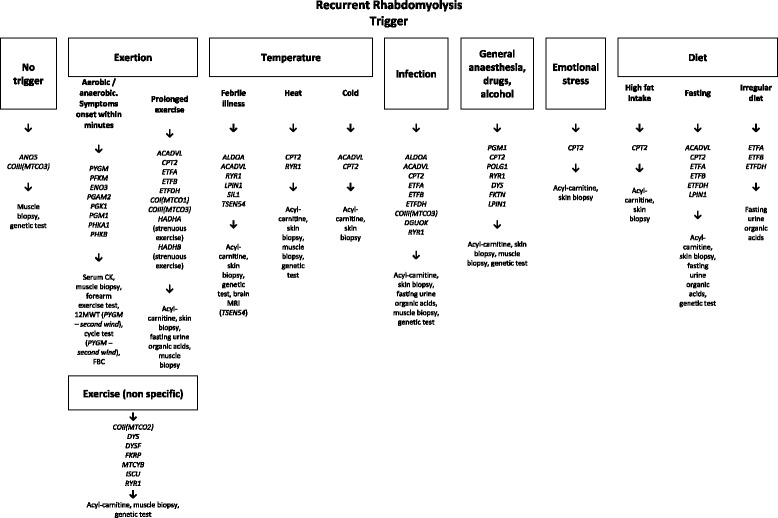
Examples of different triggers of rhabdomyolysis. The identification of triggers may help guiding genetic testing and may also aid the interpretation of variants of uncertain significance identified on next generation sequencing in patients presenting with RM. CK: creatine kinase, 12MWT: 12 minute walk test, FBC: full blood count, MRI: magentic resonance imaging.

**Figure 3:**
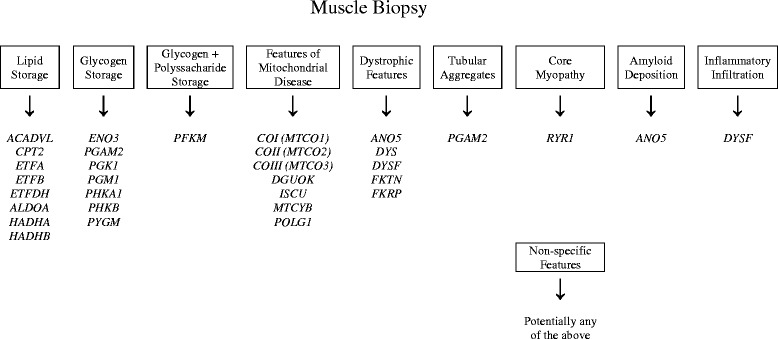
Muscle biopsy patterns associated with hereditary causes of RM. Muscle biopsy features may provide a guide to targeted genetic testing.

Identifying underlying genetic disorders presenting with RM can pose a diagnostic challenge due to their rarity and marked heterogeneity, requiring a high degree of clinical suspicion for appropriate investigation to be requested. Although difficult, establishing a genetic diagnosis in a patient presenting with RM is of great clinical importance to give appropriate advice and to prevent future episodes. More recently Zutt et al. (2014) suggested an algorithm for the clinical diagnostic approach to recurrent RM [2] whereas the present review will focus on the approach to a specific genetic diagnosis. Here we review the genetic defects known to be associated with RM, many of them are rare and very rare (summarized in Table 1). The purpose of this review is to highlight the most important features in order to aid the genetic diagnosis of patients with unexplained, in particular recurrent RM episodes (Figures 2 and 3).

**Table 1:** **Inherited neuromuscular disorders associated with episodes of rhabdomyolysis**

**Gene**	**Disease name**	**Baseline CK levels**	**Pattern of inheritance**	**Trigger for rhabdomyolysis**
**Disorders of glycogen metabolism**				
***PYGM***	Glycogen storage disease type V, McArdle disease	High	AR	Aerobic and anaerobic exercise, symptom onset within minutes
*PFKM*	Glycogen storage disease type VII, Tarui’s disease	High	AR	Aerobic and anaerobic exercise, symptom onset within minutes
*ALDOA*	Glycogen storage disease type XII	Normal	AR	Febrile illness, infection
		Mild elevation, High		
*ENO3*	Glycogen storage disease type XIII	Normal	AR	Aerobic and anaerobic exercise, symptom onset within minutes
		High		
*PGAM2*	Glycogen storage disease type X	High	AR	Aerobic and anaerobic exercise, symptom onset within minutes
*PGK1*	Phosphoglycerate kinase 1 deficiency	Normal	X-linked	Aerobic and anaerobic exercise, symptom onset within minutes
		High		
*PGM1*	Glycogen storage disease type XIV	High	AR	Aerobic and anaerobic exercise, symptom onset within minutes, general anaesthesia
*PHKA1*	Glycogen storage disease type IX	?	X-linked	Aerobic and anaerobic exercise, symptom onset within minutes
*PHKB*			AR	
**Disorders of fatty acid metabolism:**				
***ACADVL***	Deficiency of very-long-chain acyl-CoA dehydrogenase	Normal	AR	Fasting, prolonged exercise, cold, infections, fever
		High		
***CPT2***	Carnitine palmitoyl-transferase deficiency	Normal	AR	Prolonged exercise, fasting, fever, infection, high fat intake, cold exposure, heat, emotional stress, drugs
*ETFA*	Glutaric aciduria Type II	Normal	AR	Physical exercise, fasting, irregular diet or infection
*ETFB*	Multiple acyl-coenzyme A dehydrogenase deficiency	Mildly to moderately elevated		
*ETFDH*				
**Mitochondrial disorders**				
*COI (MTCO1)*	Mitochondrial disorder	Normal	Maternal inheritance	Prolonged or repetitive exercise
*COII*	Mitochondrial disorder	Normal	Maternal inheritance	Exercise
*(MTCO2)*				
*COIII (MTCO3)*	Mitochondrial disorder	Normal	Maternal inheritance	Prolonged exercise, viral illness, unknown cause
*DGUOK*	Mitochondrial disorder	?	AR	Viral illness
*FDX1L*	Mitochondrial disorder	Normal	AR	? After exercise [114]
		High		
*HADHA*	Mitochondrial Trifunctional Protein Deficiency	Normal	AR	Strenuous physical activity
*HADHB*				
*ISCU*	Iron–sulphur cluster deficiency myopathy (Mitochondrial disorder)	?	AR	Exercise
*MTCYB*	Mitochondrial disorder	Normal	?Sporadic mutations [64]	Exercise
*POLG1*	1 case report of rhabdomyolysis in association with PIS [73]		AD, AR	PIS
**Disorders of intramuscular calcium release and excitation-contraction coupling**				
***RYR1***	Malignant hyperthermia-susceptibility, Exertional rhabdomyolysis, Congenital myopathy	Normal or mildly to moderately elevated (usually <1000 IU/L)	AD, AR	Heat, infection, alcohol, drugs, anaesthetic (MHS) and exercise
**Muscular dystrophies**				
*ANO5*	Anoctaminopathy-5	High	AR	Unprovoked – no trigger has been identified
***DMD***	Duchenne muscular dystrophy, Becker muscular dystrophy	High	X-linked	Exercise, anaesthetic drugs
*DYSF*	LGMD2B, Miyoshi myopathy	High	AR	Exercise
*FKTN*	Fukuyama congenital muscular dystrophy	High	AR	One case following the use of halothane and succinylcholine [89,90]
***FKRP***	LGMD2I	High	AR	Exercise [82]
**Miscellaneous**				
***LPIN1***	Phosphatidic acid phosphatase deficiency	Normal, High	AR	Febrile illness, anaesthesia and fasting.
*SIL1*	Marinesco-Sjogren syndrome	Normal, High	AR	Febrile infection
*TSEN54*	Pontocerebellar hypoplasia type 2	Normal, High	AR	Hyperthermia

The table above summarises genes, disease name, baseline serum CK levels (between acute episodes of rhabdomyolysis), pattern of inheritance and triggers for rhabdomyolysis. Genes commonly associated with rhabdomyolysis episodes are indicated in bold.

CK: creatine kinase; AR: autosomal recessive; AD: autosomal dominant; MHS: malignant hyperthermia susceptibility; PIS: propofol infusion syndrome; LGMD: limb-girdle muscular dystrophy (2B and 2I).

### Metabolic disorders

Inherited metabolic disorders of skeletal muscle are rare conditions that can be divided into those disorders caused by abnormal glycogen storage affecting muscle and disorders of fatty acid oxidation. In metabolic myopathies, exploring patients’ symptoms in relation to the timing and type of exercise will provide a strong clinical clue. The other clue to a metabolic diagnosis is that symptoms are experienced in all skeletal muscles including, neck, jaw, arms, paraspinals and legs. In disorders of glycogenolysis (such as McArdle disease) and glycolysis (such as Tarui disease) symptoms are induced within minutes by isometric muscle contraction (such as weight lifting), intense maximal exercise (such as sprinting) or within a few minutes of aerobic physical activity (such as walking). By contrast, in disorders of fatty acid metabolism symptoms occur only after aerobic exercise for a more prolonged period (over 45 minutes and often after several hours). In addition, fever, fasting and stress can induce symptoms. Symptoms are not triggered by isometric contraction in people with fatty acid metabolism dysfunction. Thus, a detailed medical history with careful consideration of the precipitating factors is essential for the correct diagnosis to be made. However, phenotypic variations do exist, for example in glycogen storage disorder type XIV, caused by recessive mutations in *PGM1,* where additional dysmorphic and systemic features such as bifid uvula, cleft palate, liver disease and growth retardation are prominant (outlined in more detail below) [5]. Recognising both the general symptoms of metabolic myopathies and the specific phenotypic variations of specific genetic defects is essential to guide further investigations.

Thus, despite recent advances in the genetic diagnosis, a detailed targeted medical history remains the most important initial step for identifying the underlying inherited metabolic causes of RM and to aid choice of invasive diagnostic investigations such as skin (in suspected fatty acid oxidation disorders) and muscle biopsy, for tissue histochemistry and/or biochemical analysis (in suspected glycogen storage disorders). In the majority of these conditions, inheritance is autosomal-recessive, with only few conditions inherited in an X-linked, autosomal-dominant or mitochondrial fashion.

### Disorders of glycogen metabolism

A variety of enzymes are involved in glycogen metabolism, an important energy source for skeletal muscle during isometric, anaerobic exercise, aerobic glycolysis and strenuous exertion. Genetic defects leading to absence or severe deficiency of these enzymes cause impaired glycogen breakdown, resulting in exercise intolerance and RM. In addition, in some of these glycogen storage disorders (GSDs) enzymes are expressed in tissues other than skeletal muscle, leading to other clinical manifestations such as haemolytic anaemia, brain and skin involvement. A feature of these conditions is a suboptimal rise in lactate during exercise and exaggerated rise in ammonia in McArdle disease. Diagnosis is dependent upon biochemical analysis of muscle tissue which reveals the reduced enzyme activity. Diagnosis is confirmed by genetic studies.

McArdle disease (GSDV) is the most common disorder in this group, and it will therefore be reviewed in more detail. Other types of GSDs are extremely rare and their features will only be briefly summarized.

#### Muscle Glycogen Phosphorylase (PYGM)

Homozygous or compound heterozygous mutations in *PYGM* (OMIM #608455) cause glycogen storage disease type V (GSDV, McArdle disease) which is characterized by the absence of skeletal muscle glycogen phosphorylase, resulting in an inability to convert muscle glycogen into glucose-1-phosphate [6,7]. As a result, glycogen accumulates in skeletal muscle.

##### Phenotype

Exercise intolerance presenting as muscle fatigue, discomfort and cramps within minutes of exertion, commonly associated with a disproportionate increased in heart rate (HR) [8,9]. Symptoms are more severe with strenuous exercise such as climbing stairs or walking up hills and isometric contraction (heavy lifting or squatting) [10]. Prolonged muscle spasm (muscle contracture) is not uncommon and can lead to RM. The *second wind* phenomenon is characterized by symptoms easing and an associated decrease in HR leading to an improvement in exercise tolerance occurring at around 8–10 minutes of aerobic exertion. It is the hallmark of this condition and is due to improved blood flow resulting in improved availability of glucose release from the hepatic glycogen stores and fatty acid oxidation metabolism [11]. Other clinical features may include muscle hypertrophy. Fixed weakness predominantly affecting shoulder girdle and axial (paraspinal) muscles is another feature of GSDV usually seen in older patients [9,10]. GSDV symptoms usually begin during early childhood but patients often experience several episodes of myoglobinuria and RM before the diagnosis is confirmed [12].

##### Triggers for Rhabdomyolysis

Persistent skeletal muscle activity despite symptoms (before getting into the *second wind* phenomenon), intense exertion, anaerobic activity, isometric contraction and sustained muscle contracture [13,14].

##### Diagnostic approach

Serum CK is almost always raised, often >1,000 IU/L. Muscle biopsy shows subsarcolemmal vacuoles with glycogen accumulation and the absence (or virtual absence in rare cases) of muscle glycogen phosphorylase activity demonstrated by muscle histochemistry and/or muscle biochemical enzyme analysis [15]. Functional testing using either cycle test or 12 minute walk test can be used to demonstrate the presence of the *second wind* phenomenon [16]. Forearm exercise test shows suboptimal (<3 fold) increase in serum lactate with a normal rise in ammonia [17]. Non-ischaemic exercise test is preferred because of the risk of contracture and compartment syndrome in people with McArdle disease.

#### Phosphofructokinase, Muscle Type (PFKM)

Autosomal-recessive mutations in the *PFKM* gene (OMIM #610681) cause GSD type VII, or Tarui’s disease, a metabolic myopathy characterized by phosphofructokinase (PFK) deficiency resulting in impaired conversion of fructose-6-phosphate to fructose-1.6-diphosphate, affecting both anaerobic and aerobic glycolysis. In general, GSDVII shares similar presentation with GSDV (see above) except that GSDVII presents with more severe exercise intolerance.

##### Phenotype

Similar to GSDV but there is no *second wind* phenomenon. In fact, unlike GSDV where oral administration of glucose immediately prior to exercise can improve exercise tolerance, in Tarui disease it exacerbates exercise intolerance leading to an ‘*out-of-wind phenomenon’* by impairing fatty acid oxidation [18]. Atypical variants reported include severe infantile form with arthrogryposis, a form presenting with fixed myopathy and haemolytic anaemia without muscle symptoms. Hypertrophic cardiomyopathy has been reported in one patient [15,18-20].

##### Triggers for Rhabdomyolysis

Exercise and isometric muscle contraction [20].

##### Diagnostic approach

The combination of haemolytic anaemia and myopathy should raise the suspicion of GSDVII [18,20]. The combination of haemolytic anaemia and muscle symptoms can also be associated with GSDXII and phosphoglycerate kinase deficiency described below. Serum CK is usually raised and forearm exercise test reveals a suboptimal increase in lactate. As in McArdle disease, hyperuricaemia and gout are more frequent. Muscle biopsy shows a vacuolar myopathy with polyglucosan accumulation demonstrated by positive periodic acid-Schiff (PAS) staining but resistant to diastase digestion [18] and weak/negative PFK histochemistry [15,18,20]. Enzyme biochemical analysis shows reduced enzyme activity in muscle and, in some patients, in erythrocytes [20].

#### Aldolase A, Fructose-bisphosphate (ALDOA)

GSDXII or aldolase A deficiency is a very rare metabolic disorder characterized by the deficiency of the aldolase A isoform which converts fructose 1,6-biphosphate into dihydroxyacetone phosphate and glyceraldehydes 3-phosphate. As a result, glycolysis is affected in muscle and erythrocytes. GSDXII is caused by autosomal-recessive mutations in the *ALDOA* gene (OMIM #103850).

##### Phenotype

Non-spherocytic haemolytic anaemia. Rare cases with myopathic manifestations including muscle pain and weakness, exercise intolerance and RM [21,22]. RM may be seen during the first months of life [23].

##### Triggers for Rhabdomyolysis

Febrile illness [21-23] and infection [24].

##### Diagnostic approach

Reduced red blood cell aldolase in combination with high serum CK suggest the diagnosis [22,25]. Muscle biochemistry shows reduced aldolase activity [21]. The spectrum of histopathological findings in GSDXII and their clinical relevance are currently unclear. In one patient the following pathological features were reported: variation in fibre size, split fibres and increased activity of acid phosphatase. Electron microscopy (EM) revealed accumulation of lipid, increased variation in mitochondrial size and shape, variation in myofibrilar diameter and increased intermyofibrillar space [21]. The presence of lipid droplets accumulation in oil-red-O histochemistry was reported in another patient [23]. Diagnosis is confirmed by finding homozygous or compound heterozygous mutations in *ALDOA.*

#### Beta Enolase 3 (ENO3)

Autosomal-recessive mutations in the *ENO3* gene (OMIM #131370) cause GSD XIII or muscle β-enolase deficiency. β-enolase is a distal glycolytic enzyme.

##### Phenotype

Symptoms of exercise intolerance are reported to be milder than those seen in GSDV. There is no *second wind* phenomenon. Symptoms include exercise-induced myalgia and cramps [26,27].

##### Triggers for Rhabdomyolysis

Strenuous exertion and isometric muscle activity [27].

##### Diagnostic approach

CK may be normal or mildly raised between attacks of RM. Forearm exercise test shows a suboptimal increase in serum lactate [27]. Muscle biopsy examination under light microscopy may be unremarkable while EM may show glycogen accumulation [26,27]. Biochemical analysis of muscle tissue shows severely reduced activity of β –enolase [26,27]. Diagnosis is confirmed by genetic studies.

#### Phosphoglycerate Mutase 2 (PGAM2)

Autosomal-recessive mutations in the *PGAM2* gene (OMIM #612931) cause GSD type X, a metabolic myopathy characterized by the reduction of muscle phosphoglycerate mutase (PGAM) resulting in a terminal block in glycogenolysis. A mild residual activity of the brain isoform of the enzyme may be detected in muscle tissue.

##### Phenotype

Symptoms are much milder than GSDV in that symptoms are not present during most daily activities but can be precipitated by intense bursts of activity [18,28-31]. There is no *second wind* phenomenon.

##### Triggers for Rhabdomyolysis

Intense exertion such as running and strength training exercise [28,29,31,32].

##### Diagnostic approach

Serum CK is usually raised. Forearm exercise test may show a mild suboptimal raise in lactate [18,31]. Biochemical analysis of muscle tissue shows reduced enzyme activity [31]. Muscle biopsy may show tubular aggregates [28,29] and glycogen accumulation [30]. Thus the triad of exercise-induced cramps, recurrent myoglobinuria and tubular aggregates on muscle biopsy should alert the clinician to the possibility of PGAM deficiency [29].

#### Phosphoglycerate Kinase 1 (PGK1)

Phosphoglycerate kinase 1 (PGK) deficiency (OMIM #300653) is an X-linked disorder characterized by the reduction of phosphoglycerate kinase 1, which catalyses the conversion of 1,3-diphosphoglycerate to 3-phosphoglycerate.

##### Phenotype

Marked clinical variability has been described, including haemolytic anaemia, muscle cramps and myalgia, weakness, increased serum CK levels, exercise intolerance, RM and central nervous system (CNS) manifestations including mental retardation and development delay [33-36].

##### Triggers for Rhabdomyolysis

Intense exercise [33,35,36].

##### Diagnostic approach

Serum CK may be raised or within normal range. Full blood count showing reticulosis and mildly raised serum bilirubin may suggest haemolysis. Forearm exercise test shows suboptimal rise in lactate with exaggerated rise in ammonia [35,36]. Muscle biopsy findings may be unremarkable [33,36] or show minor changes such as fibre atrophy [35]. EM may reveal glycogen accumulation [35,37]. Biochemical analysis shows decreased PGK enzyme activity in muscle, erythrocytes [35-37], white cells and platelets [35].

#### Phosphoglucomutase 1 (PGM1)

Recessive mutations in *PGM1* (OMIM #171900) cause GSD type XIV or phosphoglucomutase 1 deficiency, a clinically heterogeneous multisystem disorder with features of a metabolic disorder and a congenital disorder of glycosylation [5].

##### Phenotype

A recent study reviewed the clinical phenotype of GSDXIV [5]. Reported symptoms include endocrine disorders, liver disease, bifid uvula, cleft palate, growth retardation, hypogonadic hypogonadism, hypoglycaemia and cardiomyopathy. Muscle symptoms include exercise intolerance, weakness and RM [5]. Bifid uvula and liver disease were the most common features recently reported by Tegtmeyer et al. (2014) [5].

##### Triggers for Rhabdomyolysis

Strenuous exercise [5,38]. Malignant hyperthermia and RM were reported in one patient after halothane administration [39]. The combination of propofol and remifentanil caused mild raised CK in the same patient [39].

##### Diagnostic approach

Serum CK may be raised. Serum transferrin isoforms are abnormal [5]. Incremental exercise test until exhaustion on a cycle ergometer may show normal elevation of serum lactate levels [38]. Muscle biopsy may show glycogen accumulation and reduction of phosphoglucomutase activity on biochemical evaluation [38].

#### Phosphorylase Kinase, Muscle, Alpha-1 Subunit (PHKA1), Phosphorylase Kinase, Liver, Alpha-2 Subunit (PHKA2), Phosphorylase Kinase, Beta Subunit (PHKB), Phosphorylase Kinase, Testis/Liver, Gamma-2 (PHKG2)

Mutations in the *PHKB*, *PHKA1*, *PHKA2*, *PHKG2* genes (encoded by chromosome 16, X, X and 16 respectively) cause GSD type IX, characterized by the deficiency of phosphorylase kinase (PK) resulting in impairment of glycogen metabolism. PK has four subunits differentially expressed in different tissues and encoded by different genes. Symptomatic muscle PK deficiency may be seen in association with *PHKB* (OMIM #172490) and *PHKA1* (OMIM #311870) mutations, although a mild transitory form of muscle weakness and myalgia after strenuous exertion has been recently reported in two patients with *PHKG2* mutations [40]. The phosphorylation of phosphorylase b into its active form (phosphorylase a) is catalysed by the PK [41]. Thus muscle symptoms of GSDIX shares similar symptoms with GSDV, but they are usually milder. Here we will focus on mutations in *PHKB* and *PHKA1* which are known to affect the skeletal muscle isoform.

##### Phenotype

Hepatomegaly, hypoglycemia following fasting and growth delay are the primary presenting symptoms of mutations in *PHKB* [42], although mild muscle symptoms occur. Muscle symptoms associated with mutations in *PHKA1* are usually mild and include exercise-induced myalgia and cramps, fatigue and myoglobinuria [43,44]. Raised CK following statin use was reported in association with *PHKA1* in one patient [44]*.*

##### Triggers for Rhabdomyolysis

Intense exertion (*PHKA1*) [43,45].

##### Diagnostic approach

Serum CK may be raised in between episodes of RM [46]. Muscle histology may show nonspecific abnormalities and free glycogen accumulation [43,46]. PK activity is reduced in muscle. Reduced enzyme activity in erythrocytes may be seen but normal values do not exclude the condition. Forearm exercise test may show an exaggerated increase in ammonia but a normal lactate response [44].

### Disorders of fatty acid metabolism

Lipids are an important energy source of skeletal muscle during endurance exercise, recovery following physical exertion and when at rest. Different enzymes participate in long-chain fatty acid breakdown in mitochondria. Beta-oxidation of long chain fatty acids differs from mitochondrial disorders, where primary downstream effect usually directly impair oxidative phosphorylation, ultimately reducing ATP production as described below (*mitochondrial disorders* section). Defects in the beta-fatty acid oxidation pathway lead to disorders of fatty acid metabolism, frequently characterized by exercise intolerance, muscle pain and episodes of RM related to prolonged aerobic exercise and other triggers such as fever, fasting, stress, drugs (such as sodium valproate and statins) and certain anaesthetic drugs. RM may also be related to exertion when resumed after rest following the onset of symptoms.

*Propofol Infusion Syndrome* (PIS) is a rare condition following the administration of high doses of propofol. Symptoms include a wide range of systemic complications such as cardiovascular, respiratory, metabolic, and hepatic dysfunction [47]. RM may be seen in association with PIS. Young age, high dose of propofol, concomitant administration of vasopressor and underlying critical illness are considered risk factors for PIS. Inborn errors of mitochondrial fatty acid oxidation have been listed as risk factors for PIS [47] although the pathogenic mechanism is not fully understood.

#### ACYL-CoA Dehydrogenase, Very long-chain (ACADVL)

Homozygous or compound heterozygous mutations in the *ACADVL* gene (OMIM #609575) cause a metabolic myopathy due to very long-chain acyl-CoA dehydrogenase (VLCAD) deficiency, an enzyme associated with the inner mitochondrial membrane that plays an important role in the first step of mitochondrial long chain fatty acid oxidation.

##### Phenotype

Varies in severity depending upon age of presentation and includes three main clinical presentations: 1) severe neonatal/early-childhood onset form presenting with cardiomyopathy, hepatic disease and hypotonia with high mortality in infancy, 2) milder childhood onset form with hypoketotic hypoglycaemia, hypotonia with or without hepatic disease and 3) adult-onset form presenting with exercise intolerance, muscle cramps and RM [48,49]. However, there may be some symptom overlap between the three clinical forms and RM has also been reported in the mild childhood onset form [48] which may evolve into an adult onset phenotype with age. Patients presenting with the adult-onset form may have symptoms between episodes of RM [49]. Severity and genotype may be correlated, corresponding to the mutation-dependent level of the residual enzyme activity (for example, severe early-childhood onset form with no residual enzyme activity is associated with the homozygous R429W mutation) [48].

##### Triggers for Rhabdomyolysis

Fasting, prolonged aerobic exercise, emotional stress, shivering and cold, or other catabolic stress such as infections and fever [49-51] and certain drugs such as sodium valproate and statins.

##### Diagnostic approach

Serum CK may be normal or raised between attacks and may vary considerably between separate measurements. Muscle biopsy is unhelpful and should not be performed if VLCAD is suspected, instead the most important first line investigation is fasting blood acyl-carnitine profile which shows accumulation of long-chain acyl-carnitines usually with prominent C14:1. Functional testing of fatty acid oxidation studies in skin fibroblasts is abnormal. Diagnosis is confirmed by finding homozygous or compound heterozygous mutations in *ACADVL*.

#### Carnitine Palmitoyltransferase II (CPT2)

Autosomal-recessive mutations in the *CPT2* gene (OMIM #600650) cause carnitine palmitoyl-transferase-II (CPT-II) deficiency, the most common disorder of fatty acid oxidation. CPT-II deficiency is characterized by reduced or absent enzyme activity depending on the genotype.

##### Phenotype

The clinical phenotypes are very similar to VLCAD deficiency with three main forms: 1) fatal neonatal form presenting with dysmorphic features, severe skeletal and cardiac muscle involvement, 2) infantile-onset form with hepatic, cardiac and skeletal muscle involvement with hypoketotic hypoglycemia and 3) late (juvenile or adult) onset with myalgia, exercise intolerance and recurrent RM [52-54]. Importantly, enzyme defect and a more severe phenotype have been correlated with certain genotypes (for example, homozygous p.R631C and heterozygous null mutations in *trans* with a second mutation) [53].

##### Triggers for Rhabdomyolysis

Prolonged exercise, fever, heat shock, infection, high fat intake, fasting, exposure to cold, emotional stress, drugs including sodium valproate and statins [53] and lipid soluble intravenous anaesthetic drugs [52]. A malignant hyperthermia-like syndrome was described in association with the heterozygous (R503C) mutation following succinylcholine and halothane administration [55].

##### Diagnostic approach

As with VLCAD, CK may be normal in between episodes and muscle biopsy is not helpful and should not be performed if CPT-II deficiency is suspected. Fasting blood acyl-carnitine profile may show accumulation of long-chain acyl-carnitines usually with prominent C16, C18:1, C18 and is the preferred first line investigation. Enzyme activity may be assessed in muscle tissue, platelets/leukocyte and skin biopsy (cultured fibroblasts). Diagnosis is confirmed by finding mutations in *CPT2*, however approximately 60% of affected people carry the common (c.338C > T, p.Ser113Leu): thus, testing of this specific mutation is recommended as a second line investigation [56].

#### Electron Transfer Flavoprotein, Alpha Polypeptide (ETFA), Electron Transfer Flavoprotein, Beta Polypeptide (EFTB), Electron Transfer Flavoprotein Dehydrogenase (ETFDH)

Autosomal-recessive mutations in the *EFTA* (OMIM #608053), *EFTB* (OMIM #130410) and *ETFDH* genes (OMIM #231675) cause glutaric aciduria type II (GAII or multiple acyl-coenzyme A dehydrogenase deficiency), a metabolic condition characterized by the deficiency of the alpha or beta subunits of electron transfer flavoprotein or the electron transfer flavoprotein dehydrogenase. GAII results in a metabolic disturbance usually associated with acidosis due to the inability to breakdown fatty acids (mitochondrial fatty acid oxidation) and amino acids to generate energy. Choline metabolism is also affected. As a result, metabolism and excretion of organic acids such as glutaric acid are impaired.

##### Phenotype

May vary according to age of presentation with essentially three main forms: 1) neonatal-onset form presenting with hypotonia, hepatomegaly, hypoglycaemia and metabolic acidosis in association with congenital abnormalities such as dysmorphism and polycystic kidneys and high mortality in infancy, 2) neonatal-onset form with no congenital abnormalities and 3) late-onset form presenting with a wide spectrum of symptoms including myopathy, metabolic acidosis and hypoglycaemia [52,57]. Muscle symptoms include myalgia, weakness, exercise intolerance and RM [57-61]. Muscle weakness may respond to riboflavin given orally. Cardiomyopathy may be seen in all forms [15].

##### Triggers for Rhabdomyolysis

Reported triggers for RM are physical exercise, fasting, irregular diet or infection [58]. Triggers for metabolic decompensation may also include sodium valproate therapy, weight loss, alcohol intake and febrile illness [57].

##### Diagnostic approach

Increased fasting urine organic acids (glutaric acid, ethylmalonic acid, isovaleric acid, a-methylbutyrate, isobutyrate, aliphatic dicarboxylic acids, and their derivatives) and plasma acyl-carnitine profile (increased C4-C12) may confirm the diagnosis when evaluated during a metabolic stress episode [59]. Plasma free carnitine level may be decreased [15]. Muscle biopsy may show lipid accumulation and enlarged mitochondria [15,57,58].

### Mitochondrial disorders

Mitochondria are highly dynamic organelles that provide cellular energy, in the form of adenosine triphosphate (ATP), via oxidative phosphorylation. Since skeletal muscle tissue has very high energy requirements, it is particularly sensitive to impaired mitochondrial function.

Mitochondrial diseases usually refer to genetic defects whose primary downstream effect directly impairs oxidative phosphorylation which ultimately reduces ATP production. However, a number of additional essential metabolic processes occur within mitochondria, such as beta-oxidation of long chain fatty acids. Mutations in genes encoding enzymes involved with beta-oxidation disrupt mitochondrial fatty acid oxidation and include CPT-II and VLCAD deficiency, both of which are discussed above.

The clinical presentation of mitochondrial disorders is extremely variable. This is, in part, due to the large number of genetic causes implicated in the development of disease. One important, although relatively infrequent, manifestation of mitochondrial disease is RM. Table 1 summarizes a few examples of genes associated with mitochondrial dysfunction and RM.

#### Phenotype

Multisystem features vary according to the genetic mutation and may include cardiomyopathy, liver disease, Leber hereditary optic neuropathy, bowel dysmotility, Leigh syndrome, developmental delay and mental retardation. Muscle symptoms may include myopathy, fatigue, exercise intolerance, myalgia, limb and facial weakness, recurrent RM episodes and muscle cramps [62-71].

#### Triggers for Rhabdomyolysis

Physical activity/exercise and viral illness have been associated with RM, although often it may not be possible to identify a specific trigger [64,66,67,69,71]. Mitochondrial dysfunction has been considered to play an important role in PIS (see above, *disorders of fatty acid metabolism* section), although the pathogenic mechanisms of PIS are not fully understood yet. PIS has been recently associated with Leber hereditary optic neuropathy [72] and *POLG1* [73] although the reported patients had more than one risk factor for PIS. Further studies are needed to confirm a genetic predisposition for PIS.

#### Diagnostic approach

Serum CK may be normal or raised. In one patient acyl-carnitine profile mimicked acyl-CoA dehydrogenase deficiency in *COII* mutation (m.8156dupG mutation) [71]. Raised serum lactate may be seen at rest and following exercise [63,64]. Muscle biopsy may reveal ragged red fibres, cytochrome c oxidase (COX)-negative fibres and subsarcolemmal accumulation of mitochondria. Ragged red fibres staining positive for COX and succinate dehydrogenase (SDH) may be seen in association with a cytochrome b gene defect [63,64]. Reduced SDH activity, patchy COX deficiency, iron accumulation and ultrastructurally abnormal mitochondria containing electron dense inclusions have been associated with iron-sulfur cluster assembly enzyme [15,67]. Nonspecific myopathic features may be seen. Analysis of respiratory chain enzymes may help the diagnosis. First line investigation for mtDNA common mutations may be performed in a blood sample but usually skeletal muscle is required to sequence mtDNA for cases presenting with RM.

### Disorders of intramuscular calcium release and excitation-contraction coupling

Excitation-contraction coupling (ECC), i.e. the effective translation of an electrical neuronal impulse into muscle contraction, is an intricate process involving several intramuscular ion channels and pumps. The key players involved in ECC are the voltage-gated dihydropyridine receptor localized on the transverse tubules, the ligand-gated skeletal muscle ryanodine receptor (RyR1) localized on the sarcoplasmic reticulum, and various sarcoendoplasmic reticulum (SR) calcium transport ATPases (SERCAs). Although neuromuscular phenotypes have been associated with all 3 proteins, *RYR1*-related neuromuscular disorders are by far the most common and may also feature RM episodes.

#### Ryanodine Receptor 1 (RYR1)

Mutations in the *RYR1* gene (OMIM#180901) cause a wide spectrum of neuromuscular phenotypes, ranging from the dominantly inherited MHS trait [74] to various congenital myopathies, including dominantly inherited Central Core Disease [75] and subgroups of recessively inherited Multi-minicore Disease [76], Centronuclear Myopathy [77] and Congenital Fibre Type Disproportion [78]. *RYR1* encodes the principal SR calcium release channel with a crucial role in skeletal muscle excitation-contraction (E-C) coupling.

##### Phenotype

A wide range of neuromuscular features may be seen in association with *RYR1* mutations, including hypotonia, developmental delay, facial weakness with or without ptosis and extraocular muscle involvement, axial and proximal muscle weakness. However, *RYR1*-related (exertional) rhabdomyolysis occurs mainly in individuals with MHS-associated *RYR1* mutations who rarely exhibit any muscle weakness, but rather on the contrary, may exhibit muscle hypertrophy and even superior athletic abilities. In addition to exertion-induced RM, myalgia, muscle stiffness and heat intolerance are common in this group of patients [3,75]. King-Denborough syndrome and late-onset axial myopathy are other myopathic manifestations closely associated with MHS-related *RYR1* mutations [79,80].

##### Triggers for Rhabdomyolysis

Heat, exercise, anaesthetic (MHS), muscle relaxants (MHS), drugs and alcohol ***but not*** fasting. Exercise is the most common trigger, but often a combination of factors appears to be required to trigger an episode [3,81]. In contrast to some of the metabolic conditions outlined above, RM episodes events may occur hours (occasionally days) after intense and unaccustomed exercise. Infection may unmask MHS-related *RYR1* mutations [3,4] and may be an underdiagnosed cause of RYR1-related rhabdomyolysis.

##### Diagnostic approach

Muscle pathology findings may vary. A recent review of patients presenting with rhabdomyolysis due to (mainly MHS-associated) dominant RYR1 mutation showed that muscle biopsy findings were often non-specific, but may feature “RYR1-compatible” findings including irregular internal architecture or core-like structures, increased internal nuclei, type 1 fibre predominance and pinprick fibres expressing neonatal myosin [3]. Muscle magnetic resonance imaging (MRI) findings in the same cohort often showed marked muscle hypertrophy corresponding to clinical features [3], but not the pattern of selective involvement typically seen in *RYR1*-associated congenital myopathies [4], in keeping with the divergent clinical phenotypes.

### Muscular dystrophies

Muscular dystrophies (MD) represent a clinically and genetically heterogeneous group of disorders characterized by progressive weakness and skeletal muscle degeneration. There is an increased susceptibility for muscle damage and RM may be seen in association with exertion but the timing of onset of symptoms with duration of exercise is often vague. Exertion muscle symptoms and myoglobinuria may be the presenting symptom of an underlying MD even before weakness becomes clearly manifest [82]. Becker muscular dystrophy (BMD) due to mutations in *DMD* and limb-girdle muscular dystrophy 2I (LGMD2I) due to mutations in *FKRP* (OMIM#606596) are the most common dystrophies to present with RM [82]. RM and myalgia may be the dominating symptom in a few cases of LGMD2I [82]. RM as the presenting symptom of MDs has also been reported in association with sarcoglycanopathies, dysferlinopathies [83,84] and the more recently reported conditions related to recessive mutations in *ANO5*. Recessive mutations in *ANO5* (OMIM#608662) cause a wide spectrum of myopathies including LGMD2L, distal myopathy and isolated hyperCKemia. Exercise intolerance, severe myalgia, variable muscle weakness and RM have been associated with *ANO5* [85,86], although specific triggers for RM could not be elicited [85]. Identification of amyloid deposition on muscle biopsy is a clue for the diagnosis of an anoctaminopathy [85,86]

Thus, unexplained RM occurring in the context of a history of exertion myalgia should also raise the suspicion of MDs, even if no weakness is clearly manifest. Figure 1 and Table 1 summarises the main MDs associated with RM [8,83,85-90].

### Miscellaneous

#### LPIN1

Autosomal-recessive mutations in the *LPIN1* gene (OMIM #605518) have been reported as the second most common cause of early-onset RM [91]. Impaired lipid synthesis of triglycerides and membrane phospholipids as well as energetic defect have all been hypothesised as pathogenic mechanisms for RM in this disorder, although the exact mechanism remains unclear [92].

##### Phenotype

Early onset usually before 6 years with a mean age of onset of approximately 21 months [91,92] with severe episodes of RM with high mortality [92,93]. Myalgia and stiffness may be seen during acute episodes of RM [93]. Episode frequency may decrease with age [92].

##### Triggers for Rhabdomyolysis

Fever, general anaesthesia, fasting [91,92].

##### Diagnostic approach

Between episodes serum CK and acyl-carnitine profile may be normal. Muscle biopsy can be normal or it can show lipid accumulation. Type I fibre predominance, type II fibre atrophy and ragged-red fibres have been reported [15,91,94]. Genetic testing is required to confirm the diagnosis.

#### SIL1, S. Cerevisiae, Homolog Of (SIL1)

Autosomal-recessive mutations in *SIL1* (OMIM #608005) cause Marinesco-Sjogren syndrome, a multisystem disorder with consistent neuromuscular involvement.

##### Phenotype

Cataracts, cerebellar atrophy and ataxia are usually seen in association with different multisystem signs and symptoms. Neuromuscular findings include hypotonia, development delay, weakness, a variably associated peripheral neuropathy, and, rarely, RM [15,95,96].

##### Triggers for Rhabdomyolysis

Infection [95,97].

##### Diagnostic approach

Muscle biopsy may show a broad spectrum of myopathic features including rimmed vacuoles, increase in connective tissue with fat infiltration, ragged red fibres and type I fibre predominance. EM may show unique dense membranes/tubules surrounding the muscle fibre nucleus [15].

#### tRNA Splicing Endonuclease 54, S. Cerevisiae, Homolog Of (TSEN54)

Pontocerebellar hypoplasia type 2 (PCH-2) is a neurodegenerative disorder characterized by hypoplasia and atrophy of pons and cerebellum in association with other CNS manifestations. Recent reports suggest a myopathic component in PCH-2, including RM that has been reported in association with mutations in the *TSEN54* gene (OMIM #608755).

##### Phenotype

Global developmental delay, microcephaly, epilepsy, movement disorders and other CNS manifestations may be seen. Muscle symptoms include hyperCKaemia and RM [98,99].

##### Triggers for Rhabdomyolysis

Febrile infections and hyperthermia [98,99].

##### Diagnostic approach

Brain MRI may confirm the CNS abnormalities. Muscle biopsy may be normal or show fibre atrophy on light microscopy. EM may show abnormalities including sarcomeric disruption and Z-band abnormalities even in patients where light microscopy is normal [98].

### Genetic Polymorphisms and association with RM

In addition to the genetic conditions detailed above, different genetic polymorphisms have already been associated with CK elevations, however it is not entirely clear if the same polymorphisms also confer a higher risk for RM, as contradictory findings have been reported. One example of such contradiction is the I allele in the angiotensin I-converting enzyme (*ACE*) gene which has been previously associated with increased CK levels after eccentric exercise by Yamin et al. (2007) but not by other authors [100-102]. The same has been observed with the R577X *ACNT3* variant: the XX genotype, which has been reported in athletes [103], was also associated with low muscle strength and low resting CK [104] and exertional RM [100].

These intriguing findings emphasise the complexity of RM pathophysiology and the need for further studies to clarify the role of polymorphisms in muscle breakdown. Table 2 summarises a few genetic polymorphisms previously reported in association with CK elevations and muscle symptoms [100-102,104-107].

**Table 2:** **Polymorphisms previously reported to be associated with exercise related muscle injury**

**Gene**	**Exercise related symptoms**	**rs# (dbSNP)**	**Comments**	**Ref**
*ACE*	Increased CK levels following eccentric exercise	rs4340	Dose dependent increase of CK (II higher than ID)	[102]
	No association	rs4340	-	[101]
	No association	rs4340	-	[100]
*ACTN3*	Exertion rhabdomyolysis	rs1815739	TT genotype	[100]
	No association with CK/Mb changes	rs1815739	TT genotype – lower baseline CK	[104]
*CCL2*	Exercise-induced skeletal muscle damage following maximal eccentric exercise	rs3917878, rs13900, rs1024611, rs1860189	rs3917878 – high CK response (women) and attenuated strength recovery (men) rs13900, rs1024611 and rs1860189 - abnormal preexercise CK level (women)	[106]
*CCR2*	Exercise-induced skeletal muscle damage following maximal eccentric exercise	rs3918358, rs768539, rs1799865,	rs3918358 – slower strength recovery (women) rs1799865 – soreness rs1799865 – abnormal preexercise CK level (women) rs768539 and rs3918358 – preexercise strength (men)	[106]
*CKMM*	Exertion rhabdomyolysis	rs1803285	GG genotype	[100]
	Increased CK following exertion	rs1803285	AA genotype	[101]
*IGF-II*	Muscle damage following maximal isotonic eccentric contractions	rs3213221, rs680, rs7924316, rs2132570	Strength loss, soreness and high CK (Muscle damage indicators)	[105]
*IL6*	Increased CK levels following maximal eccentric exercise	rs13447445	Dose dependent increase of CK (CC higher than CG genotypes)	[107]
	No association	rs13447445	-	[100]
*MYLK*	No association	rs2700352	-	[100]
	Muscle damage following maximal eccentric exercise	rs2700352, rs28497577	GG has greater increase in CK and myoglobin following exercise, higher baseline strength AC greater increase CK following exercise, postexercise strength loss (no AA data)	[104]
	Exertion rhabdomyolysis	rs28497577	AC or AA	[100]
*TNFA*	Increased CK levels following maximal eccentric exercise	rs361525	Mild association	[107]

CK: creatine kinase, I: insertion - *I* allele; D: deletion - *D* allele; Ref: reference.

## Conclusion

Inherited muscle disorders associated with RM are heterogeneous and rare. Much of the current knowledge concerning these conditions is based on individual case reports. Within the group of inherited muscle disorders, RM occurs most frequently in disorders of muscle metabolism (most commonly due to mutations in *ACADVL, CPT2* and *PYGM*), mitochondrial dysfunction, muscular dystrophies (most commonly seen in BMD and LGMD2I), congenital myopathies where intracellular calcium homeostasis and excitation-contraction coupling are affected (*RYR1*) and *LPIN1*. Awareness of specific genotype-phenotype correlations in genetic conditions associated with RM is of importance to inform the diagnostic approach. In individual cases, RM events may reflect a combination of a genetic predisposition and environmental triggers, and the presence of an identifiable trigger does not necessarily exclude an underlying genetic cause.

A substantial proportion of patients presenting with RM remain currently genetically unsolved, suggesting additional pathogenic mechanisms not yet discovered. Polymorphisms causing an additive effect in the pathogenesis of RM have been suggested by different authors but may prove difficult to confirm. New diagnostic techniques such as next generation sequencing and whole exome/genome sequencing may help to evaluate patients for comprehensive gene sets concomitantly as well as identifying new genetic causes of RM [108,109].

## Abbreviations

ATP: Adenosine triphosphate
BMD: Becker muscular dystrophy
CK: Creatine kinase
CNS: Central nervous system
COX: Cytochrome c oxidase
CPT-II: Carnitine palmitoyl-transferase-II
ECC: Excitation-contraction coupling
EM: Electron microscopy
GAII: Glutaric aciduria type II
GSD: Glycogen storage disorder
HR: Heart rate
LDH: Lactate dehydrogenase
LGMD2I: Limb-girdle muscular dystrophy 2I
MD: Muscular dystrophy
MHS: Malignant hyperthermia susceptibility
MRI: Magnetic resonance imaging
PAS: Periodic acid-Schiff
PCH-2: Pontocerebellar hypoplasia type 2
PFK: Phosphofructokinase
PGAM: Phosphoglycerate mutase
PGK: Phosphoglycerate kinase
PhK: Phosphorylase kinase
PIS: Propofol infusion syndrome
RM: Rhabdomyolysis
RyR1: Skeletal muscle ryanodine receptor
SDH: Succinate dehydrogenase
VLCAD: Very long-chain acyl-CoA dehydrogenase
